# A Hand Book of Medical and Surgical Reference

**Published:** 1874-08

**Authors:** 


*A Hand-Book of Medical and Surgical Reference.* By JOHN H. WYETH, M. D. New York: Wm. Wood & Co. Buffalo: H. H. Otis.

